# The role of therapeutic plasma exchange in plasma cell disorders

**DOI:** 10.1007/s00277-024-05712-0

**Published:** 2024-04-01

**Authors:** Danai Dima, Jack Khouri

**Affiliations:** Department of Hematology and Medical Oncology, Taussig Cancer Institute, Cleveland Clinic, Cleveland, OH USA

**Keywords:** Therapeutic plasma exchange, Plasma cell disorders, Myeloma cast nephropathy

## Abstract

Therapeutic plasma exchange (TPE) is an extracorporeal technique where patient’s plasma containing pathogenic substances is separated and removed from the whole blood, while the cellular component is returned to the patient mixed with replacement solution via an apheresis machine. Due to its ability to remove pathogenic substances from plasma including immunoglobulins, TPE has proven efficacious in the management of various disorders across different medical disciplines, including plasma cell dyscrasias, which are characterized by the abundant secretion of non-functional immunoglobulins produced by an abnormally proliferating plasma cell clone. This review summarizes the current indications of TPE in plasma cell-related disorders and discusses its application, safety, and therapeutic effects.

## Introduction

Therapeutic plasma exchange (TPE) is a modality that has proven efficacious in the treatment of a wide variety of disorders across different medical disciplines [1]. It involves the removal of the patient’s plasma that contains pathogenic substances which may aid in halting the disease process and potentially improving clinical outcomes. First, patient’s blood is passed through an external filter to separate plasma from cellular components. The removed plasma is discarded, and replaced with a combination of colloid and crystalloid solutions [2]. After the removal of plasma, the cellular components and replacement fluid are mixed and returned to the patient.

The most commonly targeted pathogenic substances with TPE are antibodies [2]. TPE is also believed to modulate the immune system by altering the ratio of T helper type-1 and type-2 cells in the peripheral blood, which interact with antibody-secreting B-cells ultimately impeding antibody production [3]. Because of these immunological functions, TPE has been established as an important treatment modality in patients with plasma cell disorders, which are characterized by the abundant secretion of non-functional immunoglobulins produced by an abnormally proliferating plasma cell clone [4]. In certain circumstances, high amounts of the non-functional immunoglobulin can cause various complications necessitating removal via TPE. These scenarios mainly include myeloma cast nephropathy, hyperviscosity syndrome most commonly in the setting of Waldenström macroglobulinemia, and cryoglobulinemia [5, 6]. This review summarizes the application of TPE in plasma cell-related disorders and discusses its indications and therapeutic effects in combination with other therapies.

### Myeloma cast nephropathy

Multiple myeloma (MM) is the second most common hematologic malignancy in adults accounting for 17% thereof [7]. Despite major improvement in survival with the advent of novel agents, MM remains an incurable malignancy [8]. Renal impairment is a myeloma defining event and seen in up to 50% of patients at initial presentation [9, 10]. Myeloma cast nephropathy (MCN) is the main etiology of kidney failure and accounts for 30% of all cases [11, 12]. MCN has a significant impact on patient outcomes with a median survival of less than a year in those who remain dialysis dependent. A study by Royal et al. showed a strong association between renal recovery (best glomerular filtration rate [GFR]) and overall survival with a hazard ratio of 1.6 per every 15 ml/min/1.73m^2^ drop in GRF below 45 ml/min/1.73m^2^ [13]. The pathophysiology of MCN is related to mechanisms mainly involving the monoclonal serum free light chains (sFLC). Free light chains bind to Tamm–Horsfall protein in distal tubules of the kidneys forming toxic casts that result in tubule obstruction and eventually tubular atrophy and fibrosis. In addition, free light chains induce proximal tubular damage by triggering inflammatory changes leading to tubulointersitial fibrosis [14–16]. Therefore, a swift and efficient reduction of sFLC is paramount to mitigate permanent kidney damage and improve patient survival.

Major efforts have been put into studying extracorporeal sFLC removal techniques, most commonly TPE for the treatment of MCN. Current treatment guidelines and expert opinions recommend the rapid institution of plasma cell-directed therapy and consideration of TPE, with the goal of quick reduction in the circulating sFLCs [12, 17, 18]. It has been shown that early reduction in sFLCs is associated with improved renal responses and ultimately OS [13, 19]. Previous reports have highlighted favorable outcomes of MM patients with MCN treated with bortezomib-based regimens [20–27]. Three randomized controlled trials of TPE in MCN have been published to date and they were all conducted before the introduction of novel plasma cell-directed agents [28–30]. The role of TPE in the treatment of MCN remains controversial due to inconsistent study results, study design flaws including misrepresentative endpoints, lack of biopsy confirmation, suboptimal concomitant chemotherapy and low statistical power.

The largest randomized study of TPE in MCN was published in 2005 by Clark et al., and included 97 patients with acute kidney injury, 30% of whom were dialysis-dependent [30]. It randomized patients to cytotoxic therapy consisting of melphalan/prednisone or vincristine/adriamycin/dexamethasone with or without TPE (58 versus 39 patients, respectively). The primary endpoint was a composite outcome of death, dialysis dependence and GFR < 30 ml/min at 6 months. More patients became dialysis independent in the TPE arm (66% versus 50%) but no statistical significance was reached for the difference in primary endpoint (57 versus 69%); overall survival (OS) was also similar at 6 months. The main downsides of this study were the lack of confirmatory kidney biopsies, potentially ineffective cytotoxic therapy, and the use of a composite outcome (death, dialysis dependence or GFR < 30 at 6 months) that may have hindered the efficacy of TPE in improving renal outcomes. The study also performed a fixed number of TPE sessions (5–7) and did not follow sFLC level measurements for guidance on number of required TPE treatments; thus, some patients may have been undertreated.

A small, prospective study by Johnson et al. randomized 21 patients to receive TPE plus chemotherapy versus chemotherapy alone. TPE in combination with chemotherapy reduced the sFLC more rapidly than chemotherapy alone. Notably, all three out of the 12 patients in the TPE plus chemotherapy arm who required dialysis, were able to recover their renal function. However, overall renal recovery and overall survival rates were similar between the two arms [28]. The study was criticized for being underpowered. A retrospective study from Mayo Clinic looked at the role of TPE in 40 patients with MM and renal failure and reported a renal response rate of 45% [25]. In addition, the study found a correlation between renal recovery (defined as decrease by half in initial serum creatinine and dialysis independence) and the degree of sFLC improvement with TPE (a drop by at least half) in patients with biopsy-confirmed MCN (75% renal recovery) [25]. Therefore, implementing TPE in only those patients who have biopsy-proven MCN and targeting at least a 50% drop in sFLC may ensure renal recovery and dialysis independence. A meta-analysis of the three above studies showed a significantly lower 6-month dialysis dependence with TPE than chemotherapy alone (15.6% versus 37.2%; risk ratio, 2.02; *p* = 0.04) [31]. Again, no difference in OS was noted. Other retrospective studies have found improved renal outcomes with TPE along with bortezomib-based plasma cell-directed therapy in MCN patients as long as a significant reduction in sFLC is achieved [24, 26].

**Fig. 1:**
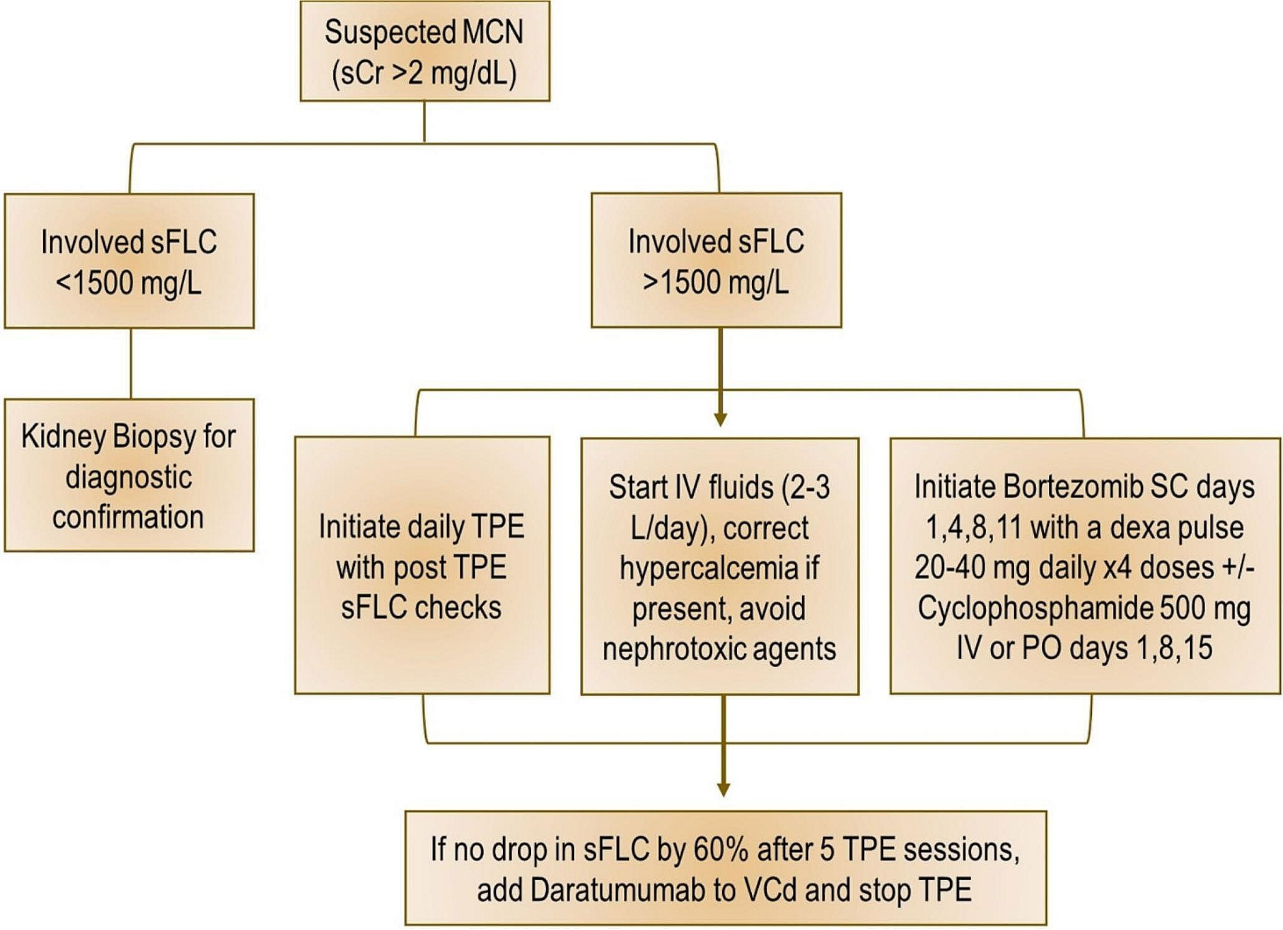
Management of Myeloma Cast Nephropathy. **Abbreviations**: MCN, myeloma cast nephropathy; sCR, serum creatinin, sFLC, serum free light chains; TPE, therapeutic plasma exchange; SC, subcutaneous; IV, intravenous; PO, oral; dexa, dexamethasone; VCd, bortezomib, cyclophosphamide, dexamethasone

Despite the controversial role of TPE in the treatment of MCN, the International Myeloma Working Group (IMWG) and American Society of Apheresis (ASFA) do recommend TPE as adjunctive therapy along with plasma cell-directed therapy [18, 32]. Our current approach for patients with newly-diagnosed MM and MCN is summarized in Fig. 1. For patients with sFLC > 1500 mg/L, we start plasma-cell directed therapy with bortezomib, cyclophosphamide and dexamethasone (VCd) along with supportive care, daily TPE and serial sFLC checks. If a drop in sFLC by 60% is not achieved after 5 TPE sessions, we favor adding the anti-CD38 antibody daratumumab (dara) to VCd and stopping TPE to ensure rapid light chain drop and avoid removal of dara by the apheresis machine. We avoid administering dara simultaneously with TPE due to drug removal by the machine which may hinder its efficacy. For patients with relapsed-refractory MM and MCN, TPE is considered on a case-by-case basis. Future prospective studies should evaluate novel plasma cell-directed therapies with or without TPE for patients with MCN to better delineate the role of TPE for the treatment of MCN especially in the contemporary era of frontline daratumumab use.

### Type I cryoglobulinemia

Cryoglobulinemia (cryo) is a systemic disorder characterized by the presence of cryoglobulins, which are immunoglobulins that precipitate at cold temperatures [33]. Cryoglobulins may be symptomatic or incidental, and can be monoclonal or polyclonal. They are classified into three categories depending on the type of cryoglobulin isotype and underlying process driving its production and pathogenesis. Type I cryo is usually symptomatic and typically treated by hematologists as it is driven by monoclonal immunoglobulins, most commonly of IgG or IgM isotypes produced by a plasma cell or B cell clone, respectively [33, 34]. The clone is benign in most cases as is in monoclonal gammopathy of undetermined significance but could also be malignant (myeloma, chronic lymphocytic leukemia, or Waldenstrom’s macroglobulinemia) [33–35]. Notably, severe disease tends to be associated with the IgG isotype more commonly than IgM [33, 35]. In type II cryo, the cryoglobulins are a combination of monoclonal IgM with rheumatoid factor activity and polyclonal IgG. It is usually driven by hepatitis C infection but has been also seen in a variety of autoimmune disorders, other infections, and B cell malignancies (not plasma cell disorders). Polyclonal IgM and IgG are the hallmark of type III cryo, typically seen in the setting of infections including hepatitis C, and autoimmune disorders. The following section will discuss the treatment of type I cryo and the role of TPE.

In type I cryo, the clinical manifestations are related to the aggregation of cryoglobulins in small blood vessels leading to obstruction and end-organ damage. This occurs at cold temperatures but could also take place at higher temperatures if the cryoglobulins are present at high concentration. The skin is the most frequently involved organ based on multiple studies including one with 102 patients with type I cryo; peripheral sites (distal extremities, nose and ears) tend to be mostly involved given the associated lower temperatures of those anatomical structures [34, 36, 37]. Purpura and skin ulcers are most common, cyanosis and livedo reticularis are also prevalent [33, 36, 37]. Peripheral neuropathy is present in about a third of the patients, mostly sensory followed by Raynaud’s phenomenon and arthralgias in a quarter of the patients, respectively [34]. Renal involvement is uncommon (14% of patients) and chiefly presents as membranoproliferative glomerulonephritis (MPGN) with proteinuria with or without tubular dysfunction [34]. The gastrointestinal and respiratory tracts, and central nervous system are typically spared in type I cryo [33, 36, 37].

The treatment of type I cryo is generally directed at the underlying lymphoproliferative clone. Thus, clone specific therapy is the mainstay of treatment for most patients. There are no randomized clinical trials that have addressed the role of TPE in the treatment of type I cryo. TPE is effective at reducing circulating cryoglobulin burden and potentially improve symptoms if the latter are indeed related to cryo. Thus, it could be implemented when urgent removal of cryoglobulins is required, typically due to severe disease (such as glomerulonephritis, skin ulcerative disease, and debilitating neuropathy) as shown in case series and cohort studies [33–37]. TPE has a category II indication for the treatment of severe cryo of any type as per ASFA guidelines recommending its use as a second-line treatment, either as monotherapy or in conjunction with other systemic therapies including clone-directed therapies [32]. In addition, TPE could be helpful as sole therapy in frail patients who may not tolerate cytotoxic agents or patients whose disease became refractory to clone-directed therapies.

Our approach at Cleveland Clinic is in line with the ASFA recommendations. In general, we perform daily or every other day one plasma-volume exchanges for a total of five treatments with subsequent clinical and lab evaluation. The frequency of TPE is tailored to each individual patient; we consider maintenance therapy for some patients to help prevent symptom recurrence especially those with advanced renal and neurological involvement [32]. The cryocrit is the volume of the cryoglobulin precipitate relative to the serum volume and tends to be higher in type I as compared to other cryo types, and dropss with TPE. However, it does not correlate with disease severity [32, 33]. Therefore, it is generally not considered a reliable biomarker in deciding when to discontinue TPE. Instead, we typically rely on symptomatology, organ response (especially in kidney and nerve) and disease-specific hematologic response to guide therapy duration.

### Hyperviscosity syndrome

Hyperviscosity syndrome (HVS) is a complication of elevated immunoglobulins mostly in the setting of monoclonal disorders [38], including MM, Waldenstrom’s macroglobulinemia (WM), and cryoglobulinemia. In detail, HVS can be seen in 10–30% of patients with WM associated with IgM paraprotein, and less frequently in MM (2–6%), where it is mainly associated with IgA paraprotein isotype [38, 39]. Symptoms of HVS are variable ranging from vision impairment, headache, dizziness or mucosal bleeding to seizures and respiratory failure. In patients with WM, HVS symptomatology typically occurs when IgM levels reach above > 4000 mg/dL and plasma viscosity exceed 4 cp. although it can certainly occur at lower levels. In IgA MM, the paraprotein threshold associated with HVS is usually higher, up to > 6000–7000 mg/dL [39].

Due to HVS being potentially life-threatening, prompt diagnosis and treatment are critical. When symptomatic hyperviscosity is present, TPE should be immediately started for paraprotein removal along with systemic therapy targeting the underlying malignant cells. Duration of treatment is directed by symptoms and daily or every other day TPE is recommended until symptom improvement [40]. Commonly, 1–3 TPE sessions are sufficient [39]. For patients with WM and IgM levels > 4000 mg/dL, prophylactic TPE should be performed prior to receiving rituximab, even in the absence of HVS symptoms, as rituximab can cause a transient IgM increase or “IgM flare” related to its rapid release from the necrotic cancer cells causing emergence or deterioration of existing symptoms [40]. In these cases, a single TPE session is most often adequate before systemic therapy initiation. Notably, IgM is a mostly intravascular molecule and levels drop remarkably fast with a reduction of blood viscosity from 30 to 70% with each TPE treatment. In some cases, maintenance TPE therapy may be considered to help avoid symptom reemergence especially in patients whose malignancy has become refractory to clone-directed therapies.

### Technical notes

At Cleveland Clinic, we use the Spectra Optia® Apheresis System for our TPE procedures. We use citrate (ACD-A) for circuit anticoagulation. The use of anticoagulants is essential to minimize blood coagulation in the extracorporeal circuit. TPE is well-tolerated overall, the main potential side effects are hypocalcemia, fluid shifts and hypotension, vasovagal syncope, transfusion reactions, coagulopathy (hypofibrinogenemia) and electrolyte imbalances [41]. We perform one plasma volume exchanges and typically use albumin 5% and normal saline for fluid replacement. Plasma is used in certain patients with coagulopathies, bleeding, low fibrinogen, recent surgical procedures or certain disorders that require plasma to address the underlying physiology such as thrombotic thrombocytopenic purpura. We administer prophylactic calcium replacement during the treatment and supplement as needed if symptoms of hypocalcemia develop.

## Conclusion

TPE is a useful modality in the symptomatic management of a variety of complications associated with plasma cell dyscrasias. It does not address the underlying pathology but can effectively and rapidly remove the pathologic paraprotein, thus temporarily relieving symptoms or reversing organ damage. In the majority of cases, it must be combined with therapy directed against the underlying clone for effective long-term control and prevention of symptom recurrence.

## Data Availability

No datasets were generated or analysed during the current study.
